# Validation of a Population-Based Data Source to Examine National Cancer Clinical Trial Participation

**DOI:** 10.1001/jamanetworkopen.2022.3687

**Published:** 2022-03-22

**Authors:** Angela K. Green, Sara M. Tabatabai, Xing Bai, Akriti Mishra Meza, Anne-Marie Lesny, Carol Aghajanian, Ola Landgren, Gregory J. Riely, Paul Sabbatini, Andrew Salner, Scott Lipkin, Andrew Ip, Peter B. Bach, Colin B. Begg, Sham Mailankody, Allison Lipitz-Snyderman

**Affiliations:** 1 Department of Medicine, Memorial Sloan Kettering Cancer Center, New York, New York; 2 Department of Epidemiology and Biostatistics, Memorial Sloan Kettering Cancer Center, New York, New York; 3 Patient Revenue Support, Memorial Sloan Kettering Cancer Center, New York, New York; 4 Myeloma Program, Sylvester Comprehensive Cancer Center, University of Miami, Miami, Florida; 5 Hartford Healthcare Cancer Institute, Hartford Hospital, Hartford, Connecticut; 6 Miami Cancer Institute, Baptist Health South Florida, Miami; 7 Division of Outcomes and Value Research, John Theurer Cancer Center, Hackensack University Medical Center, Hackensack, New Jersey; 8 Hackensack Meridian School of Medicine, Nutley, New Jersey

## Abstract

**Importance:**

The Centers for Medicare & Medicaid Services requires health care organizations to report the National Clinical Trial (NCT) identifier on claims for items and services related to clinical trials that qualify for coverage. This same NCT identifier is used to identify clinical trials in the ClinicalTrials.gov registry. If linked, this information could facilitate population-based analyses of clinical trial participation and outcomes.

**Objective:**

To evaluate the validity of a linkage between fee-for-service (FFS) Medicare claims and ClinicalTrials.gov through the NCT identifier for patients with cancer enrolled in clinical trials.

**Design, Setting, and Participants:**

This cohort study included 2 complementary retrospective analyses for a validation assessment. First, billing data from 3 health care institutions were used to estimate the missingness of the NCT identifier in claims by calculating the proportion of known participants in cancer clinical trials with no NCT identifier on any submitted Medicare claims. Second, the Surveillance Epidemiology and End Results-Medicare data set, which includes a subset of all FFS Medicare beneficiaries for whom health insurance claims are linked with cancer registry data, was used to identify adult patients diagnosed with cancer between 2006 and 2015 with an NCT identifier in claims corresponding to an interventional cancer clinical trial. To estimate the accuracy of the NCT identifier when present, the proportion of NCT identifiers that corresponded to trials that were aligned with the patients’ known primary or secondary diagnoses was calculated. Data were analyzed from March 2020 to March 2021.

**Exposures:**

An NCT identifier present in Medicare claims.

**Main Outcomes and Measures:**

The main outcome was participating in a clinical trial relevant to patient’s cancer diagnosis.

**Results:**

A total of 1 171 816 patients were included in analyses. Across the 3 participating institutions, there were 5061 Medicare patients enrolled in a clinical trial, including 3797 patients (75.0%) with an NCT identifier on at least 1 billing claim that matched the clinical trial on which the patient was participating. Among 1 171 816 SEER-Medicare patients, 29 138 patients (2.5%) had at least 1 claim with a value entered in the NCT identifier field corresponding to 32 950 unique patient-NCT identifier pairs. There were 26 694 pairs (81.0%) with an NCT identifier corresponding to a clinical trial registered in ClinicalTrials.gov, of which 10 170 pairs (38.1%) were interventional cancer clinical trials. Among these, 9805 pairs (96.4%) were considered appropriate.

**Conclusions and Relevance:**

In this cohort study, this data linkage provided a novel data source to study clinical trial enrollment patterns among Medicare patients with cancer on a population level. The presence of the NCT identifiers in claims for Medicare patients participating in clinical trials is likely to improve over time with increasing adherence with the Centers for Medicare & Medicaid Services mandate.

## Key Points

**Question:**

What are the accuracy and reliability associated with using National Clinical Trial (NCT) identifiers to link fee-for-service Medicare claims and the ClinicalTrials.gov database to identify patients with cancer in clinical trials?

**Findings:**

This cohort study including 1 171 816 patients with cancer linking Medicare claims and ClinicalTrials.gov identified 5061 Medicare patients in interventional cancer clinical trials, among whom 96% had a cancer diagnosis appropriate to the trial. Based on billing data from 3 institutions, approximately 75% of patients enrolled in interventional cancer trials had at least 1 claim with the appropriate NCT identifier.

**Meaning:**

These findings suggest that NCT identifiers could be used to link Medicare claims with ClinicalTrials.gov, creating a national registry of older adult patients treated in clinical trials.

## Teaser

This cohort study evaluates the validity linking fee-for-service Medicare claims and ClinicalTrials.gov through National Clinical Trial identifiers for patients with cancer enrolled in clinical trials.

## Introduction

Changes in the Centers for Medicare & Medicaid Services (CMS) billing requirements for patients in clinical trials have created a timely opportunity to assess the potential for a novel population-based resource to study clinical trial participation and outcomes. Based on an executive order issued in 2000, CMS reimburses for routine services associated with qualifying clinical trials for Medicare beneficiaries.^1,2^

Beginning in 2014, CMS required health care organizations to report the National Clinical Trial (NCT) identifier for items and services related to clinical trials that qualify for coverage in Medicare.^3,4^ Medicare is a federal insurance program for adults ages 65 years and older and others with qualifying conditions.^5^ The same NCT identifier is used to identify clinical trials in ClinicalTrials.gov, a US National Library of Medicine registry of clinical trials. This resource contains details of clinical trials sponsored by the National Institutes of Health (NIH), other federal agencies, and nonprofit and private organizations, including cancer clinical trials. ClinicalTrials.gov is considered a comprehensive and widely used source of clinical trial information, given that registration of clinical trials on this website is required by both the International Committee of Medical Journal Editors for publication and by the US Food and Drug Administration (FDA) for regulated drugs, biologics, or devices. ClinicalTrials.gov is populated with granular details regarding trial interventions, phase, sponsor, recruitment status, eligibility criteria, start and completion date, and outcomes.^6^

In principle, a database linking Medicare claims with the clinical trial information available on ClinicalTrials.gov through the NCT identifier could provide a rich resource to track Medicare beneficiaries’ enrollment in cancer clinical trials as well as their health care utilization and outcomes. Such a resource would be invaluable for providing population-based information regarding long-term knowledge gaps about the practice of clinical trials and disparities and inequities in cancer clinical trial enrollment, which are complex and inadequately characterized,^7,8,9^ as well as toxic effects, hospitalizations, costs, and longer-term survival measures associated with clinical trial participation. Limited data are available on the proportion of patients enrolled in cancer clinical trials and measurement in real time is a prerequisite to the analysis and refinement of interventions aimed to improve trial representativeness. However, valuable output from this resource will only be possible if the record linkage is successful and the data are comprehensive.

Therefore, we conducted a feasibility study to examine the linkage of fee-for-service (FFS) Medicare claims with ClinicalTrials.gov through the unique NCT identifier to capture patients with cancer registered in clinical trials. From 2014 to 2016, there were 54 to 57 million beneficiaries enrolled in Medicare overall^10^; approximately 69% to 70% of them were enrolled in the FFS Medicare program, in contrast to the Medicare Advantage program.^11^ A focus on cancer was appropriate for this investigation owing to the availability of the Surveillance Epidemiology and End Results (SEER)-Medicare data set, which serves as our primary validation data set. This data set contains a subset of the full FFS Medicare claims data set, linking population-based incident cases of cancer with FFS Medicare claims for patients residing in regions covered by the NCI-sponsored SEER program. Therefore, this data set permits access to important clinical information about patients beyond what would be available from their claims alone. The purpose of this study was to examine the quality of the data linkage and its validity and the breadth of data available for future research purposes.

## Methods

For this cohort study, our use of the SEER-Medicare database was deemed exempt from institutional review board review by Memorial Sloan Kettering Cancer Center because the database contains deidentified data, informed consent was not possible. Billing claims analysis was approved by the institutional review board at Memorial Sloan Kettering Cancer Center. The study followed the Strengthening the Reporting of Observational Studies in Epidemiology (STROBE) reporting guideline.

We tested the validity of the linkage using 2 distinct approaches to estimate the accuracy of the NCT identifier when present and its missingness in claims data. First, we used billing and administrative data from 3 health care institutions to estimate the assiduousness with which hospitals actually record the NCT identifier on relevant claims by calculating the proportion of known participants in cancer clinical trials who did not have the NCT identifier on any of their submitted Medicare claims. These data were used to estimate the proportion of the total Medicare patients in clinical trials whom we captured through the presence of the NCT in claims. Second, we used the SEER-Medicare data set to determine, among patients with an NCT identifier that corresponded to a known cancer clinical trial, the proportion for which the clinical trial matched the patient’s cancer type. This approach was used to determine the reliability associated with the 8-digit NCT identifier for representing the actual trial in which a patient is enrolled.

### Missingness of NCT Identifier in Billing Claims

To estimate the extent to which NCT identifiers were missing on submitted Medicare claims, we analyzed billing and administrative data obtained from 3 health care institutions. We included a convenience sample including 1 academic medical center in New York and 2 community-based institutions in Connecticut and Florida. We had planned to include additional institutions; however, owing to logistical reasons and resource availability, they were unable to generate analyzable data for the purposes of this study.

We identified patients known to have participated in an interventional cancer clinical trial and with FFS Medicare or Medicare Advantage insurance coverage. Patients who initially consented to a clinical trial but were excluded during screening or withdrew consent before receiving treatment were excluded. Billing identifiers for Medicare patients participating in clinical trials were extracted from the earliest date in which claims could be captured electronically through the institution’s current software platform to the end of follow-up for each institution. Billing claims for each patient were examined for the presence of any value entered in the NCT identifier field during the time frame the patient was enrolled in a trial. It is possible that a patient was enrolled in more than 1 clinical trial during the analysis period. If no value ever appeared in the NCT identifier field in the patient’s claims, the patient was classified as *not present*. If the value entered in the NCT identifier field matched at least 1 NCT identifier for a trial in which the patient was enrolled, then it was classified as *present–matched*. If the value entered did not match an NCT identifier, then it was classified as *present–unmatched*.

At 1 institution, there was a failure of the electronic health record billing system to automatically transfer the NCT identifier from the clinical trial management system. All records were manually checked and corrected prior to billing. Ultimately the glitch in automated NCT number transfer was corrected via reprogramming the electronic health record to clinical trial management system interface. Our analysis focused on obtaining the proportion of Medicare beneficiaries enrolled in interventional cancer clinical trials who had 1 or more billing claims with the appropriate NCT identifier (missingness of NCT identifier in billing claims).

### Accuracy of NCT Identifier in FFS Medicare Claims

#### Data Sources

We used the SEER-Medicare data set for this component of the study. The National Cancer Institute–sponsored SEER program operates registries that collect and report information about cancer incidence, characteristics, treatment, and mortality across 18 distinct geographic regions and captures approximately 30% of the US population. For patients included in the SEER program with FFS Medicare coverage, data from SEER are linked to the patient’s claims. FFS Medicare claims provide additional data for diagnoses, health services utilization, and death. Compared with the US older adult population, patients included in the SEER-Medicare data set have a similar age and sex distribution, a slightly higher proportion of people living in urban and high-income areas, and a smaller proportion of individuals who identified as non-Hispanic and non-White, such as Black, American Indian or Alaskan Native, Asian or Pacific Islander, or Hispanic individuals.^12^ While linkage to ClinicalTrials.gov does not require SEER registry, we chose to use the SEER-Medicare data source to validate the linkage between FFS Medicare and ClinicalTrials.gov because it provides more detailed data about cancer staging and diagnosis than are available in Medicare FFS.

We used data from 2006 through 2016. The requirement for the NCT identifier to be included in claims did not take effect until 2014. Therefore, our search for the NCT identifier in claims began in 2014 and ended with the latest data we had available. We chose to include patients who were diagnosed from 2006 to include both patients with newly diagnosed cancer as well as those with recurrent cancer on or after 2014.

The Aggregate Analysis of ClinicalTrials.gov (AACT) was used to obtain information about the clinical trials. AACT is a publicly available relational database that contains all information, including protocol and result data elements, about each trial registered in ClinicalTrials.gov. Although the registration of phase 1 clinical trials with ClinicalTrials.gov is not required by the FDA Amendments Act of 2007, we expect most phase 1 cancer trials are included, given the International Committee of Medical Journal Editors requirement of public registration as a condition for publication.^13^ The protocol data in AACT describe the trial characteristics, including type of intervention (when relevant), participant eligibility criteria, anticipated enrollment, trial design, and outcome measures. The results data elements include information for participant flow, baseline characteristics, outcome results, and frequencies of serious and other adverse events.

#### Patient Sample and Study Period

We identified SEER-Medicare patients ages 18 years and older with a new diagnosis of 1 of the following cancers between 2006 and 2015: bladder, breast, colorectal, head and neck, kidney, lung, non-Hodgkin lymphoma, liver or intrahepatic bile duct, melanoma, pancreas, prostate, stomach, thyroid, and uterus. Cancer diagnoses were ascertained based on the SEER registry categorization of primary tumor site.^14^ Race and ethnicity data were populated from self-reported data from the Social Security Administration. This variable was included in the comparison of demographic characteristics by presence of an NCT identifier in claims. We excluded patients without a known diagnosis date and those whose reported date of diagnosis was at or after the time of death. Men with a sole diagnosis of breast cancer were also excluded, as most clinical trials of breast cancer do not include men. To be included in the study cohort, we required that the patient had at least one National Claims History (NCH) claim during our study period of January 1, 2014, through December 31, 2016.

#### Clinical Trial Characteristics

Using AACT, we extracted the clinical trial title and the trial type (interventional, observational, observational (registry), and/or expanded access). Clinical trials were classified as interventional or observational and as cancer trials or noncancer trials (eAppendix in the Supplement). A clinical trial was classified as observational if its trial type from AACT was either observational or observational (patient registry); the trial was classified as interventional otherwise. Within interventional trials, we classified trials as cancer-related or not cancer-related based on keywords that appeared in the trial title corresponding to each NCT. An investigator (A.G.) manually checked accuracy of automated clinical trial classification for 2 cancer types (breast and lung cancer). For any clinical trials that could not be categorized based on trial title alone, we manually reviewed the trial on ClinicalTrials.gov.

### Statistical Analysis

Our analyses focused on addressing the following question: for patients with matched NCT billing claims indicating enrollment in interventional clinical trial in the SEER-Medicare data set, what proportion had a cancer diagnosis that matched the clinical trial description (ie, accuracy of NCT identifier in billing claims)?

Analyses were performed using SAS software version 9.4 (SAS Institute). The analyses were performed by a biostatistician (X.B.) and validated by a second biostatistician (A.M.M.) to ensure code reproduction. Data were analyzed from March 2020 to March 2021.

## Results

### Missingness of NCT Identifier in Billing Claims

There were 5061 adult patients with FFS Medicare or Medicare Advantage coverage who were enrolled in a clinical trial across the 3 participating institutions (academic medical center: 4850 patients; community-based institutions: 181 patients and 30 patients) (Table 1). A total of 3797 patients (75.0%) had an NCT identifier submitted on the patient’s Medicare billing claim that matched to a clinical trial in which the patient was participating (academic medical center: 3622 patients [74.7%]; community-based institutions: 145 patients [80.1%] and 30 patients [100%]). A small number of patients at the academic medical center (130 patients [2.6%]) had an NCT identifier on the billing claim but it did not correspond to any of the trials for which the patient was enrolled during the analysis period.

**Table 1.** Participants in Cancer Clinical Trials With Medicare Insurance Coverage Among a Sample of Health Care Institutions: Missingness Analysis

Cancer hospital	Analysis period^a^	Patients in interventional cancer clinical trials, No.^b^	Patients, No. (%)		
			NCT Present		NCT not present^c^
			Match^c^	Nonmatch^c^	
1	Apr 2018-Feb 2021	4850^d^	3622 (74.7)	130 (2.7)	1085 (22.4)
2	Feb 2017-Sept 2020	181	145 (80.1)	0	36 (19.9)
3	Oct 2018-Sept 2020	30	30 (100)	0	0

Abbreviation: NCT, National Clinical Trial.

^a^ Defined as the period when billing data was analyzed for the presence of the NCT in patients actively enrolled in clinical trials.

^b^ Excludes patients who enrolled in a clinical trial but were deemed ineligible in screening or withdrew consent before receiving treatment.

^c^ Presence was determined as whether there were ever data in the NCT identifier field in a patient claim. Not present indicates no NCT ever in NCT identifier field; present match, NCT in identifier field matched at least 1 NCT identifier for a trial in which the patient was enrolled; and present nonmatch, NCT in identifier field does not match any NCT identifiers for a trial in which the patient was enrolled.

^d^ Includes 13 patients with no claims submitted during the time of clinical trial enrollment.

### Accuracy of NCT Identifier in Billing Claims

We first identified 2 395 260 SEER-Medicare patients with a new diagnosis of 1 of 14 included cancer types between 2006 and 2015. While these cancer types cover most patients with cancer, they do not include all patients with cancers. We excluded 1 208 558 patients without at least 1 NCH claim submitted between 2014 and 2016, 13 147 patients with a reported cancer diagnosis at or after the time of death, and 1739 men with a sole diagnosis of breast cancer. Overall, there were 1 171 816 FFS Medicare patients in our sample. A total of 29 138 patients (2.5%) had at least 1 claim with a number entered in the NCT identifier field, corresponding to 32 950 unique patient–NCT identifier pairs. Of these, 1858 pairs (5.6%) had an NCT identifier categorized as unknown (ie, 00000000 or 99999999) and 4398 pairs (13.3%) had an 8-digit identifier that did not match with any trial in ClinicalTrials.gov. We therefore had 26 694 patient–NCT identifier pairs (81.0%) with an NCT identifier that corresponded to a clinical trial in ClinicalTrials.gov. Of these pairs, 10 170 (38.1%) were interventional, cancer clinical trials (Figure). Among all patient–NCT identifier pairs corresponding to an interventional cancer clinical trial, 9805 pairs (96.4%) were categorized as appropriate based on the patient’s known cancer diagnoses (primary or additional diagnoses) (Table 2).

**Figure.**
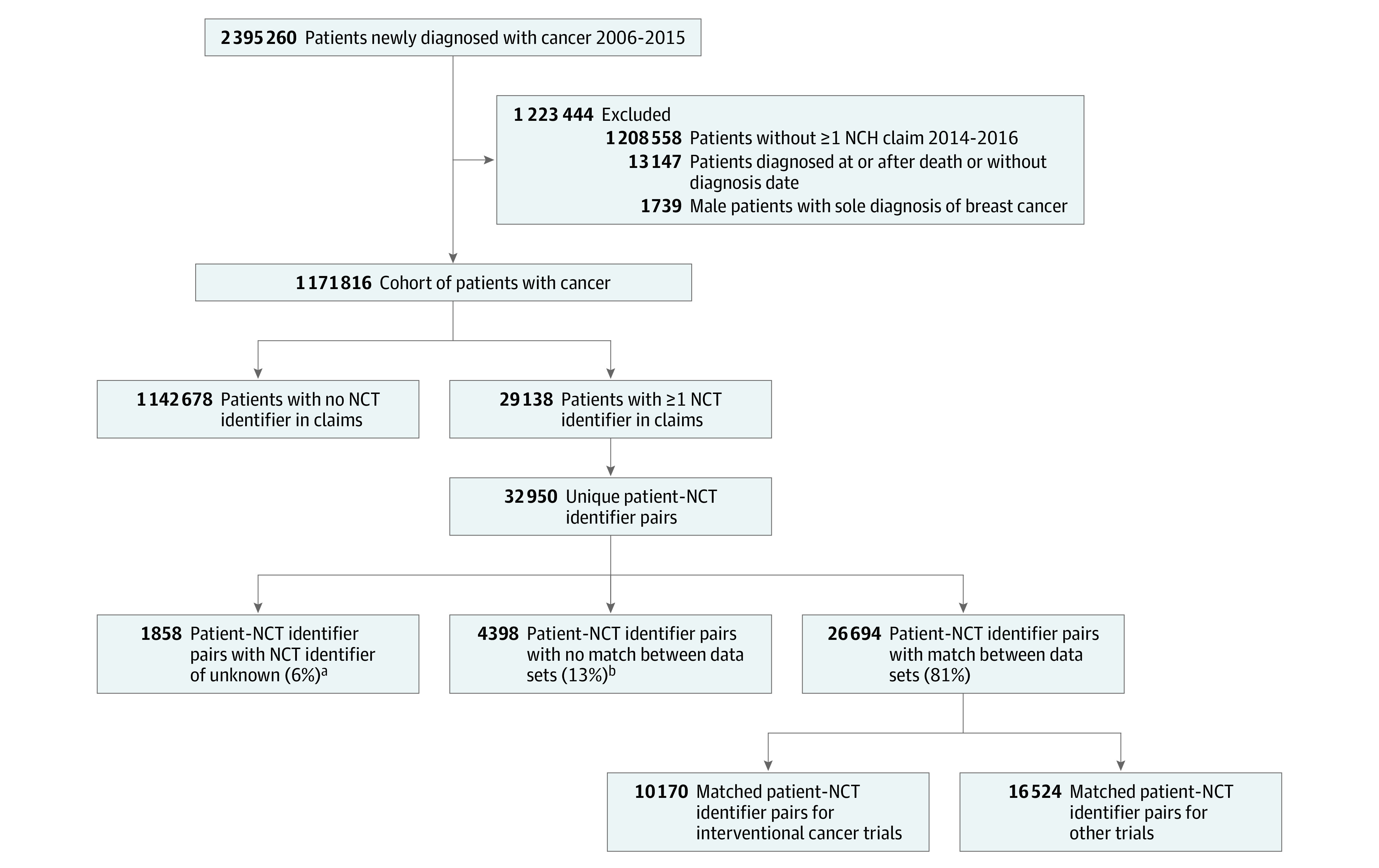
Flowchart for Surveillance Epidemiology and End Results–Medicare Patients Included in Analysis NCH indicates National Claims History. ^a^We identified unknown as National Clinical Trial (NCT) identifiers of 99999999. ^b^An NCT match was defined as an NCT identifier that had an associated trial in ClinicalTrials.gov.

**Table 2.** Patient-NCT Identifier Pairs Categorized as Appropriate Based on Primary or Secondary Diagnoses Overall and by Patient Cancer Type^a^

Cancer type	Patient-NCT identifier pairs for interventional, cancer trials, No.	Patient-NCT identifier pairs matched to cancer type, No. (%)^b^
Bladder	638	615 (96.4)
Breast	1439	1391 (96.7)
Colorectal	688	670 (97.4)
Head and neck	553	526 (95.1)
Kidney	649	627 (96.6)
Liver or bile duct	298	295 (99.0)
Lung	2214	2171 (98.1)
Non-Hodgkin Lymphoma	1093	1078 (98.6)
Melanoma	1003	954 (95.1)
Pancreas	641	630 (98.3)
Prostate	2532	2463 (97.3)
Stomach	143	134 (93.7)
Thyroid	151	135 (89.4)
Uterus	267	255 (95.5)
Overall^c^	10 170	9805 (96.4)

Abbreviation: NCT, National Clinical Trial.

^a^ For interventional, cancer clinical trials, based on SEER-Medicare data set.

^b^ Within each cancer type grouping, an NCT identifier was considered matched if the patient’s cancer diagnosis matched with the disease type eligibility criteria of the clinical trial or if the patient had multiple cancer diagnoses. Site agnostic or “basket” trials were considered appropriate for patients diagnosed with any cancer type.

^c^ Patients could contribute to multiple cancer type rows if they had more than 1 cancer diagnosis in the Surveillance Epidemiology and End Results database; 71 901 patients (6.1%) had more than 1 cancer diagnosis. The overall estimate is at the unique patient-NCT identifier level and therefore is not the sum of all cancer types.

Among 1 171 816 patients included in this analysis, cancer types ranged from 261 696 patients with prostate cancer to 16 195 patients with liver and intrahepatic bile duct cancer (Table 3; eTable 1 in the Supplement). Among these patients, 71 901 patients (6.1%) had more than 1 cancer diagnosis. Of these, 26 008 patients (2.2%) had only 1 unique NCT identifier, while 2593 patients (0.2%) had 2 NCT identifiers and 537 patients (<0.1%) had 3 or more NCT identifiers (Table 3). The median (IQR) percentage of patients across cancer types with at least 1 unique NCT identifier present was 2.5% (2.4%-3.1%). Patients with pancreatic cancer were the most likely to have at least 1 NCT identifier (800 of 20 361 patients [3.9%]), while patients with colorectal cancer (2478 of 128 870 patients [1.9%]) or uterine cancer (891 of 44 757 patients [2.0%]) were among the least likely to have at least 1 NCT identifier present in SEER-Medicare claims.

**Table 3.** SEER-Medicare Patients With Values Present on NCT Identifier Field in Claims

Cancer type	Patients, No.	No. of unique NCT identifiers listed across beneficiary’s claims, No. (%)			
		0	1	2	≥3
Bladder	76 537	74 593 (97.5)	1714 (2.2)	192 (0.3)	38 (<0.1)
Breast	256 972	251 259 (97.8)	5294 (2.1)	356 (0.1)	63 (<0.1)
Colorectal	128 870	126 392 (98.1)	2228 (1.7)	205 (0.2)	45 (<0.1)
Head and neck	45 044	43 912 (97.5)	1002 (2.2)	103 (0.2)	27 (0.1)
Kidney	50 929	49 432 (97.1)	1311 (2.6)	152 (0.3)	34 (0.1)
Liver or bile duct	16 195	15 700 (96.9)	418 (2.6)	64 (0.4)	13 (0.1)
Lung	120 702	116 916 (96.9)	3271 (2.7)	411 (0.3)	104 (0.1)
Non-Hodgkin Lymphoma	64 064	61 991 (96.8)	1762 (2.8)	260 (0.4)	51 (0.1)
Melanoma	119 053	116 112 (97.5)	2617 (2.2)	267 (0.2)	57 (<0.1)
Pancreas	20 361	19 561 (96.1)	693 (3.4)	87 (0.4)	20 (0.1)
Prostate	261 696	255 086 (97.5)	5939 (2.3)	568 (0.2)	103 (<0.1)
Stomach	16 250	15 864 (97.6)	353 (2.2)	33 (0.2)^a^	NA
Thyroid	25 898	25 270 (97.6)	576 (2.2)	40 (0.2)	12 (<0.1)
Uterus	44 757	43 866 (98.0)	804 (1.8)	75 (0.2)	12 (<0.1)
Overall^b^	1 171 816	1 142 678 (97.5)	26 008 (2.2)	2593 (0.2)	537 (<0.1)

Abbreviations: NA, not applicable; NCT, National Cancer Trial.

^a^ This cell was combined to represent patients with 2 or more unique NCT identifiers owing to small sample size.

^b^ Patients could contribute to multiple cancer type rows if they had more than 1 cancer diagnosis; 71 901 patients (6.1%) had more than 1 cancer diagnosis. The overall estimate is at the unique patient level and therefore is not the sum of all cancer types.

Patient characteristics differed by presence of an NCT identifier (Table 4; eTable 2 in the Supplement). Compared with all other patients, a notably higher proportion of patients with identifiers that matched cancer interventional trials were men (3413 patients [59.6%] vs 432 245 patients [52.3%]), in the youngest age category of 65 to 75 years (4400 patients [76.9%] vs 468 537 patients [56.7%]), diagnosed with stage 4 disease (1918 patients [33.5%] vs 87 111 patients [10.5%]), married (3884 patients [67.9%] vs 440 915 patients [53.3%]), and in the highest income quartile (3333 patients [58.2%] vs 386 528 patients [46.8%]) (Table 4).

**Table 4.** Patient Demographic and Disease Characteristics for Surveillance Epidemiology and End Results–Medicare Patients Age 65 Years and Older by Presence of NCT Identifier in Claims

Characteristic	No. (%)	
	Patients with at least 1 interventional cancer trial NCT identifier (n = 5724)	All other patients (n = 826 734)^a^
Sex		
Men	3413 (59.6)	432 245 (52.3)
Women	2311 (40.4)	394 489 (47.7)
Race		
Asian	214 (3.7)	27 084 (3.3)
Black	259 (4.5)	67 314 (8.1)
White	4904 (85.7)	689 787 (83.4)
Other or unknown^b^	347 (6.1)	42 549 (5.1)
Age at diagnosis, y		
65-74	4400 (76.9)	468 537 (56.7)
75-84	1227 (21.4)	277 017 (33.5)
≥85	97 (1.7)	81 180 (9.8)
Rurality		
Metropolitan	5207 (91.0)	711 428 (86.1)
Nonmetropolitan or unknown	517 (9.0)	115 306 (13.9)
AJCC stage		
0	263 (4.6)	114 522 (13.9)
1	883 (15.4)	239 399 (29.0)
2	1226 (21.4)	241 777 (29.2)
3	1164 (20.3)	87 166 (10.5)
4	1918 (33.5)	87 111 (10.5)
Unknown	270 (4.7)	56 759 (6.9)
Marital status		
Married	3884 (67.9)	440 915 (53.3)
Not married or unknown	1840 (32.1)	385 819 (46.7)
Geography		
Midwest	479 (8.4)	90 089 (10.9)
Northeast	1361 (23.8)	174 108 (21.1)
South	970 (16.9)	189 791 (23.0)
West	2914 (50.9)	372 746 (45.1)
Median income, $		
2512-37 916.5	374 (6.5)	103 986 (12.6)
>37 916.5 to 47 424	674 (11.8)	135 219 (16.4)
>47 424-60 430	1205 (21.1)	179 352 (21.7)
>60 430-250 014	3333 (58.2)	386 528 (46.8)
Unknown	138 (2.4)	21 649 (2.6)
Charlson Comorbidity Index score		
0	2501 (43.7)	339 900 (41.1)
1	793 (13.9)	141 683 (17.1)
≥2	532 (9.3)	131 366 (15.9)
Missing^c^	1898 (33.2)	213 785 (25.9)

Abbreviations: AJCC, American Joint Committee on Cancer; NCT, National Cancer Trial.

^a^ This category includes patients with a single NCT of 00000000 or 99999999, patients with no NCT matching between data sets, and patients who only had NCT matches between data sets for noninterventional or noncancer trials. A more detailed demographic table describing each of these categories separately is presented in eTable 2 in the Supplement.

^b^ Includes Hispanic, American Indian, and patients with race not otherwise classified.

^c^ The patients had no continuous Medicare A or B coverage or had health maintenance organization coverage from 1 year prior to through 1 month prior to their first diagnosis, which resulted in missing claims to calculate Charlson Comorbidity Index score for these patients.

## Discussion

 In this cohort study, we first assessed the completeness of reporting NCT identifiers in billing claims for Medicare beneficiaries across 3 institutions, and we found that 75% of participants in cancer clinical trials had at least 1 claim with the correctly matched NCT number. Next, we demonstrated the feasibility of linking the NCT identifiers found on FFS Medicare claims to the ClinicalTrials.gov database, and then connected 2.5% of patients with cancer diagnosed between 2006 and 2015 to interventional cancer clinical trials between 2014 to 2016. Of patients with an identified linkage to an interventional cancer clinical trial, most linkages (96%) were valid ie, the cancer diagnosis of the patients matched the conditions studied in the trial. Since 2014, CMS has required NCT identifiers on claims for clinical trial participants with Medicare insurance, thus creating an opportunity to study clinical trial participation among Medicare beneficiaries. Until now, the reliability and completeness of NCT identifiers on Medicare claims has not been assessed, to our knowledge.

We found a pattern of NCT identifier inclusion on submitted Medicare claims for patients ages 65 years and older that parallels reported disparities in clinical trial participation more generally. Specifically, patients with cancer who were younger, men, residing in zip codes with higher incomes, and with fewer comorbidities were more likely to have NCT identifiers for an interventional cancer clinical trial on their claims, consistent with prior studies^15,16,17,18^ that these patients are more likely to participate in clinical trials.

Taken together, these results suggest that the NCT identifiers included on Medicare FFS claims are sufficiently sensitive and specific to enable evaluation of the participation of Medicare patient with cancer in clinical trials. The resource may allow for near real-time tracking of clinical trial participation, which could enable stakeholders to better determine the success of cancer delivery interventions and policy decisions aimed at improving diversity and accrual. It could also be used to verify commonly cited concerns regarding cancer clinical trial enrollment, such as pervasive disparities by age, race, and ethnicity. Prior studies assessing these disparities are largely outdated and omit data regarding industry-sponsored studies, which constitute an increasing proportion of clinical trials today.^15,19^ More recent estimates on clinical trial participation rely on survey data.^20,21,22^

### Limitations

This study has some limitations. First, we assessed missingness of NCT identifiers from just 3 institutions. While these institutions included academic and community-based practices in 3 different states, they may not fully reflect reporting patterns nationally. Approximately 25% of patients on clinical trials did not have a billing claim with the appropriate NCT identifier, so we could not identify specific reasons for the missing NCT identifiers, and it is not clear if there are potential differences between patients with appropriate NCT identifiers and those without.

Second, of 32 950 unique patient–NCT identifier pairs identified, approximately 19% had an NCT identifier that did not match a valid clinical trial (observational or interventional) in ClinicalTrials.gov, a finding that may be explained by input error in the 8-digit field as well as the transitions year in 2014 when CMS allowed an NCT identifier of unknown (eg, 99999999) to be entered. However, we are unable to determine what proportion of these patients may have been in an interventional cancer trial. Future efforts to better understand issues leading to these input errors and effort to minimize these would further improve the utility of this data source. For patients with an NCT identifier indicating enrollment in interventional trials, we verified that a high proportion of trials (96%) were an appropriate match for the patient’s cancer diagnoses. We did not examine the corresponding validity of matches for noninterventional trials. While the accuracy of the NCT, when present, was excellent, the proportion of submitted claims for known clinical trial patients with Medicare insurance is less robust.

Third, as we only assessed data on select cancer types, we are uncertain to what extent our findings would generalize to clinical trials of other cancers or disease areas. However, we expect it to be reasonably uniform, given that the billing requirements are the same. Furthermore, this database captures Medicare beneficiaries in the FFS program alone, and the extent to which it is generalizable to older adults with commercial insurance is uncertain. Recent data suggest that Medicare Advantage enrollees do not differ significantly from Medicare FFS enrollees in terms of their age, race, income, and chronic conditions.^23^ However, Medicare Advantage patients are more likely to be in metropolitan areas and less likely to be in long-term care facilities.^23^ To date, there is currently no requirement for commercial insurance claims to include NCT identifiers; therefore, we do not know the extent to which clinical trial participation among patients with cancer and with commercial insurance can be identified. Thus, this data source does not inform trial participation in patients with private insurance including most individuals younger than 65 years.

## Conclusions

This cohort study found that linkage between Medicare FFS data set and ClinicalTrials.gov was feasible and could identify a proportion of older adult patients enrolled in cancer clinical trials in SEER regions that was consistent with prior estimates. This linkage provides a novel data source to study clinical trial enrollment patterns among Medicare patients with cancer on a population level. To date, there is no other resource to measure clinical trial participation in the US both comprehensively, inclusive of industry and federally sponsored studies, and longitudinally over time. As shown here, although the reporting was not complete, the current linkage has potential to be used to understand patient- and health care institution–specific characteristics that are associated with clinical trial enrollment and to highlight possible demographic, regional, and socioeconomic factors that lead to disparities in clinical trial participation. Further regulatory efforts to enhance the accuracy and completeness of reporting could augment the utility of this unique data source. Such data could inform the design of interventions to promote accrual and clinical trial diversity, a stated priority by the National Academies and the FDA.^24^

## Notes

Supplement. **eAppendix.** Coding of Clinical Trials as Cancer vs Noncancer **eTable 1.** NCT Identifiers Associated With Interventional Clinical Trials Present on Claims for Patients in the SEER-Medicare Dataset **eTable 2.** Patient Demographic and Disease Characteristics for SEER-Medicare Patients Ages 65 Years and Older by Presence of NCT Identifier in Claims, Detailed Categories
