# Supplementary material for: Validation of a Population-Based Data Source to Examine National Cancer Clinical Trial Participation
Source: JAMA Netw Open. 2022 Mar 22;5(3):e223687. doi: 10.1001/jamanetworkopen.2022.3687 (PMC8941352; doi:10.1001/jamanetworkopen.2022.3687)
Supplement: Supplement. — eAppendix. Coding of Clinical Trials as Cancer vs Noncancer eTable 1. NCT Identifiers Associated With Interventional Clinical Trials Present on Claims for Patients in the SEER-Medicare Dataset eTable 2. Patient Demographic and Disease Characteristics for SEER-Medicare Patients Ages 65 Years and Older by Presence of NCT Identifier in Claims, Detailed Categories [file jamanetwopen-e223687-s001.pdf]

Supplemental Online Content

Green AK, Tabatabai SM, Bai X, et al. Validation of a population-based data source to examine national cancer clinical trial participation. *JAMA Netw Open*. 2022;5(3):e223687. doi:10.1001/jamanetworkopen.2022.3687

eAppendix. Coding of Clinical Trials as Cancer vs Noncancer
eTable 1. NCT Identifiers Associated With Interventional Clinical Trials Present on Claims for Patients in the SEER-Medicare Dataset
eTable 2. Patient Demographic and Disease Characteristics for SEER-Medicare Patients Ages 65 Years and Older by Presence of NCT Identifier in Claims, Detailed Categories

This supplemental material has been provided by the authors to give readers additional information about their work.

© 2022 Green AK et al. *JAMA Network Open.*

eAppendix. Coding of Clinical Trials as Cancer vs Noncancer

Within interventional cancer trials, we created an algorithm to classify trials as cancer-related or not cancer-related based on the study title associated with each NCT. We created a list of cancer-related keywords (e.g. cancer, carcinoma, malignant) and a list of keywords related to common non-cancer trials (e.g. heart, cardio, pacemaker). A clinician manually categorized each trial as cancer or non-cancer for two sites (breast and lung) and percent agreement was calculate between the manual and automated classification among trials that could be categorized through the automated method (breast: 99%; lung: 100%). Following this validation, the automated system was used to classify the trials as cancer and non-cancer in the remaining sites. Trials that could not be categorized through the automated method (due to no keywords appearing in the study title) were manually categorized.

We used the variable 'clin_tril' to identify the NCT identifier on the claim. Blank 'clin_tril' variables were coded as '00000000.' We defined 'entered number' as any number in the 'clin_tril' variable other than '00000000.' Unknown = '99999999'.

© 2022 Green AK et al. *JAMA Network Open.*

eTable 1. NCT Identifiers Associated With Interventional Clinical Trials Present on Claims for Patients in the SEER-Medicare Dataset

Cancer Type	Total number of patients	Patients on an interventional, cancer trial	Patients with 1 unique interventional, cancer NCT identifier	Patients with 2 unique interventional, cancer NCT identifiers	Patients with 3+ unique interventional, cancer NCT identifiers
**Bladder**	76,537	499	428	52	19
**Breast**	256,972	1237	1129	90	18
**Colorectal**	128,870	560	486	60	14
**Head and Neck**	45,044	473	420	53[a]	--
**Kidney**	50,929	526	450	61	15
**Liver and intrahepatic bile duct**	16,195	248	209	39[a]	--
**Lung**	120,702	1838	1600	200	38
**Non-Hodgkin Lymphoma**	64,064	925	801	110	14
**Melanoma**	119,053	826	723	87	16
**Pancreas**	20,361	566	517	49[a]	--
**Prostate**	261,696	1528	1365	138	25
**Stomach**	16,250	122	109	13[a]	--
**Thyroid**	25,898	119	104	15[a]	--
**Uterus**	44,757	220	194	26[a]	--

[a]These cells were combined to represent patients with 2+ unique NCT identifiers due to small sample size

© 2022 Green AK et al. *JAMA Network Open.*

eTable 2. Patient Demographic and Disease Characteristics for SEER-Medicare Patients Ages 65 Years and Older by Presence of NCT Identifier in Claims, Detailed Categories

	No trial number present	Unknown trial number	Invalid trial number	Interventional, cancer trial number	Non-interventional or non-cancer trial number
**Total**	811,070	704	2,779	5,724	12,181
**Sex**					
Male	423,564 (52.2%)	440 (62.5%)	1,321 (47.5%)	3,413 (59.6%)	6,920 (56.8%)
Female	387,506 (47.8%)	264 (37.5%)	1,458 (52.5%)	2,311 (40.4%)	5,261 (43.2%)
**Race**					
Asian	26,720 (3.3%)	17 (2.4%)	56 (2.0%)	214 (3.7%)	291 (2.4%)
Black	66,214 (8.2%)	37 (5.3%)	165 (5.9%)	259 (4.5%)	898 (7.4%)
White	676,292 (83.4%)	612 (86.9%)	2,465 (88.7%)	4,904 (85.7%)	10,418 (85.5%)
Other[b]/Unknown	41,844 (5.2%)	38 (5.4%)	93 (3.3%)	347 (6.1%)	574 (4.7%)
**Age at Diagnosis**					
65-74	460,074 (56.7%)	484 (68.8%)	1,412 (50.8%)	4,400 (76.9%)	6,567 (53.9%)
75-84	271,237 (33.4%)	192 (27.3%)	1,079 (38.8%)	1,227 (21.4%)	4,509 (37.0%)
>=85	79,759 (9.8%)	28 (4.0%)	288 (10.4%)	97 (1.7%)	1,105 (9.1%)
**Rurality**					
Metro	697,029 (85.9%)	634 (90.1%)	2,699 (97.1%)	5,207 (91.0%)	11,066 (90.8%)
Non-metro/Unknown	114,041 (14.0%)	70 (9.9%)	80 (2.9%)	517 (9.0%)	1,115 (9.2%)
**AJCC Stage**					
0	112,256 (13.8%)	56 (8.0%)	462 (16.6%)	263 (4.6%)	1,748 (14.4%)
1	234,619 (28.9%)	141 (20.0%)	904 (32.5%)	883 (15.4%)	3,735 (30.7%)
2	237,081 (29.2%)	191 (27.1%)	815 (29.3%)	1,226 (21.4%)	3,690 (30.3%)
3	85,658 (10.6%)	115 (16.3%)	223 (8.0%)	1,164 (20.3%)	1,170 (9.6%)
4	85,835 (10.6%)	160 (22.7%)	162 (5.8%)	1,918 (33.5%)	954 (7.8%)
Unknown	55,621 (6.9%)	41 (5.8%)	213 (7.7%)	270 (4.7%)	884 (7.3%)
**Marital Status**					
Married	432,153 (53.3%)	443 (62.9%)	1,469 (52.9%)	3,884 (67.9%)	6,850 (56.2%)
Not married/ Unknown	378,917 (46.7%)	261 (37.1%)	1,310 (47.1%)	1,840 (32.1%)	5,331 (43.8%)
**Geography**					

© 2022 Green AK et al. *JAMA Network Open.*

	No trial number present	Unknown trial number	Invalid trial number	Interventional, cancer trial number	Non-interventional or non-cancer trial number
Midwest	89,101 (11.0%)	42 (6.0%)	15 (0.5%)	479 (8.4%)	931 (7.6%)
North East	168,591 (20.8%)	121 (17.2%)	2,350 (84.6%)	1,361 (23.8%)	3,046 (25.0%)
South	186,436 (23.0%)	100 (14.2%)	249 (9.0%)	970 (16.9%)	3,006 (24.7%)
West	366,942 (45.2%)	441 (62.6%)	165 (5.9%)	2,914 (50.9%)	5,198 (42.7%)
**Median Income**					
$2,512 - $37,916.5	102,153 (12.6%)	49 (7.0%)	166 (6.0%)	374 (6.5%)	1,618 (13.3%)
$37,916.5 - $47,424	133,249 (16.4%)	74 (10.5%)	214 (7.7%)	674 (11.8%)	1,682 (13.8%)
$47,424 - $60,430	176,517 (21.8%)	156 (22.2%)	321 (11.6%)	1,205 (21.1%)	2,358 (19.4%)
$60,430 - $250,014	377,869 (46.6%)	408 (58.0%)	2,029 (73.0%)	3,333 (58.2%)	6,222 (51.1%)
Unknown	21,282 (2.6%)	17 (2.4%)	49 (1.8%)	138 (2.4%)	301 (2.5%)
**Charlson Comorbidity Score**					
0	334,199 (41.2%)	285 (40.5%)	1,040 (37.4%)	2,501 (43.7%)	4,376 (35.9%)
1	138,488 (17.1%)	124 (17.6%)	630 (22.7%)	793 (13.9%)	2,441 (20.0%)
2+	127,632 (15.7%)	78 (11.1%)	699 (25.2%)	532 (9.3%)	2,957 (24.3%)
Missing[a]	210,751 (26.0%)	217 (30.8%)	410 (14.8%)	1,898 (33.2%)	2,407 (19.8%)

[a]The patients had no continuous Medicare A/B coverage or had HMO coverage from 1 year prior to through 1 month prior to their first diagnosis, which results in missing claims to calculate CCI for these patients
[b]Includes Hispanic, Native American and patients with race not otherwise classified.

Note: Patients on multiple clinical trials were assigned to a group using the following hierarchy:

- Patients with at least one NCT matching between datasets (interventional & cancer trial)
- Patients with at least one NCT matching between datasets (non-interventional / non-cancer trial)
- Patients with invalid trial number, i.e. no NCT matching between datasets
- Patients with unknown trial number, i.e. Single NCT of ‘99999999’
- Patients with no trial number present, i.e. Single NCT of ‘00000000’

© 2022 Green AK et al. *JAMA Network Open.*
